# Advances in Atypical FT-IR Milk Screening: Combining Untargeted Spectra Screening and Cluster Algorithms

**DOI:** 10.3390/foods10051111

**Published:** 2021-05-18

**Authors:** Lukas Spieß, Peter de Peinder, Harrie van den Bijgaart

**Affiliations:** 1 Qlip B.V., P.O. Box 119, 7200 AC Zutphen, The Netherlands; bijgaart@qlip.nl; 2 VibSpec, Haaftenlaan 28, 4006 XL Tiel, The Netherlands; info@vibspec.com

**Keywords:** Fourier-transform infrared, spectroscopy, milk, adulteration, spectra, untargeted, cluster, chemometrics, machine learning

## Abstract

Fourier-transform mid-infrared spectrometry is an attractive technology for screening adulterated liquid milk products. So far, studies on how infrared spectroscopy can be used to screen spectra for atypical milk composition have either used targeted methods to test for specific adulterants, or have used untargeted screening methods that do not reveal in what way the spectra are atypical. In this study, we evaluate the potential of combining untargeted screening methods with cluster algorithms to indicate in what way a spectrum is atypical and, if possible, why. We found that a combination of untargeted screening methods and cluster algorithms can reveal meaningful and generalizable categories of atypical milk spectra. We demonstrate that spectral information (e.g., the compositional milk profile) and meta-data associated with their acquisition (e.g., at what date and which instrument) can be used to understand in what way the milk is atypical and how it can be used to form hypotheses about the underlying causes. Thereby, it was indicated that atypical milk screening can serve as a valuable complementary quality assurance tool in routine FTIR milk analysis.

## 1. Introduction

Fourier-transform mid-infrared spectrometry (FT-IR) is a recognized and widely used method to rapidly determine the compositional quality of raw milk and other liquid milk products. With the use of sophisticated multivariate statistical models, it is possible to calculate from the FT-IR spectrum the concentration of fat, protein, lactose, urea, fatty acid groups, individual fatty acids [1,2,3], and other milk characteristics such as pH and freezing point. FT-IR spectrometry is also an attractive technology to screen for possible adulteration of liquid milk products [4,5,6]. In fact, various papers have been published on how infrared spectroscopy can be used to screen spectra for atypical milk composition. 

Approaches for screening atypical FT-IR milk spectra can be classified into targeted and untargeted methods [4]. Targeted methods rely on mathematical models trained to detect the presence—or estimate the quantity—of specific adulterants in the milk. Development of these models requires the adulteration of a sufficiently large and representative collection of milk samples with an adulterant, possibly at different concentrations. Due to the characteristic effect of the adulterant on the milk’s FT-IR spectrum, mathematical models can be trained to distinguish spectra belonging to adulterated milk from those belonging to normal milk. This way, mathematical models have been developed to identify milk adulterated with melamine [7], urea [3], water, starch, sodium citrate, formaldehyde, sucrose, and other adulterants [8,9]. The advantage of targeted methods is that they can indicate the presence of specific adulterants in the milk. Moreover, mathematical models tuned to specific adulterants typically exhibit lower detection limits (i.e., are more sensitive) compared to untargeted methods. The main disadvantage of targeted methods is that they are only capable of detecting known adulterants. In reality, however, it is often not known which adulterants are currently used or will be used in the future. Adulteration with substances (or complex blends thereof) that the mathematical models were not explicitly trained to detect can therefore go undetected.

This is where untargeted screening methods excel. These methods do not rely on mathematical models trained to detect a specific deviation in the FT-IR spectrum. Instead, they are sensitive to any deviation present in the spectrum by creating a mathematical model based on FT-IR spectra from authentic milk samples that still contain all normal variation (e.g., seasonal variation, different cow breeds and farm management practices). This mathematical model acts as a normal FT-IR milk fingerprint that can be compared with spectra from milk samples that are to be examined. If a spectrum deviates above a stated threshold from the normal milk fingerprint, it is denoted atypical. Since untargeted methods only rely on regular milk spectra, their development often is less expensive, and only requires a little statistical fine-tuning while offering a broad protection for possible adulteration of milk samples. This has made untargeted screening methods a popular subject of scientific research [6,10] and commercial applications [11]. Although untargeted screening methods are capable of revealing any type of adulteration with a strong effect on the spectrum, their disadvantage is that they do not comprehensibly characterize in what way a spectrum is atypical and what the root cause is. 

However, information about how and why a milk sample is atypical is crucial for effective and appropriate follow-up action (e.g., contacting the farm or manufacturer, identifying the root cause, and evaluating the possible risks for safety and quality of dairy products). In this paper, we therefore evaluate the potential of combining untargeted methods for atypical spectra screening with cluster algorithms to reveal in what way an individual milk spectrum is atypical and what the underlying cause might be. To do so, untargeted spectra screening was applied to a large dataset of bovine herd bulk milk spectra. This way, a dataset of atypical milk spectra was created. Cluster algorithms were then used to identify possible categories of atypical milk spectra. We show how information in the spectra (e.g., the compositional milk profile) and meta-data associated with their acquisition (e.g., time of measurement) can be used to understand in what way the spectrum is atypical and how it can be used to form hypotheses about the underlying causes.

## 2. Materials and Methods

### 2.1. Data Acquisition and Preprocessing

We consider a main dataset of 5,847,603 spectra that correspond to bovine herd bulk milk samples of 16,898 farms across the Netherlands. All milk samples were collected between January 2018 and November 2020 and routinely analyzed for milk payment purposes. We made sure that farmers delivering Jersey milk, having elevated fat and protein content as compared to milk from other common breeds in the Netherlands, were not present in our database. For acquisition of the spectra, milk samples were randomly assigned to one of four FT-IR instruments (Milkoscan FT +, FOSS Analytical A/S, Hillerød, Denmark) where they all underwent the same pre-treatment before the scan took place. FT-IR spectra were obtained in the mid infrared region with wavelengths between 1.995 (5012 cm^−1^) and 10.8 µm (926 cm^−1^). All spectra contained 1060 data points and were converted from transmission to absorbance. FT-IR instruments were standardized monthly using the FOSS equalizer application in accordance with the manufacturer’s instructions. Details about the standardization procedure can be found in a white paper provided by the manufacturer [12].

For each milk sample, FT-IR-based predictions of fat, protein, lactose, urea, freezing point, and milk fat acidity were available. Calculations of fat, protein, and lactose were also validated. This was done by having per batch of 47 milk samples an additional pilot milk sample with known values of fat, protein, and lactose. In addition to the spectra and the compositional milk profile, the dataset also contained meta-data concerning the time at which the FT-IR measurement took place, an identifier corresponding to the particular infrared instrument from which the spectrum was obtained, and an indicator that allowed us to determine which spectra belonged to the same farm. Basic preprocessing of the spectra consisted of the selection of relevant wavenumbers (between 925 and 1600 cm^−1^, 1690 and 1900 cm^−1^, and 2700 and 2971 cm^−1^) and a calculation of the spectra’s first derivative. Data preprocessing, analysis, and visualizations were performed using Python (version 3.7) [13] with the packages SciPy (version 1.6.0) [14], scikit-learn (version 0.23.2) [15], NumPy (version 1.20.1) [16], and Matplotlib (version 3.3.4) [17].

### 2.2. Mathematical Models

#### 2.2.1. Untargeted Spectra Screening

For the development of an untargeted mathematical model to identify atypical milk spectra, we followed a conceptually similar approach as described in [6] employed by commercial manufacturers of FT-IR instruments [11]. For the current dataset, we randomly sampled from the main dataset up to 15,000 milk spectra per month in the period between January 2018 and December 2019. This resulted in a dataset of 354,537 spectra containing twice the seasonal variation in milk. Before working with the spectral data, we first removed all spectra where the freezing point of the corresponding milk samples was above or below the highest and lowest 99 th percentile, respectively. This was done in an attempt to make the screening model more sensitive to spectral deviations indirectly associated with variations in freezing point. 

**Figure 1:**
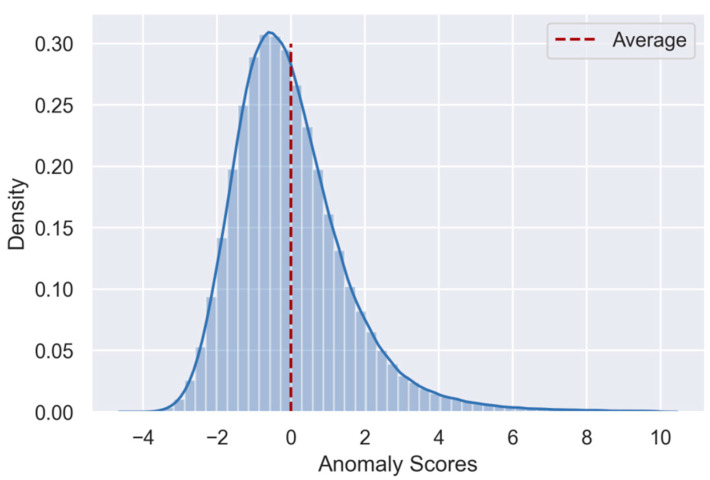
Histogram and kernel density estimate of the anomaly scores. Considered are the milk spectra used to develop the untargeted spectra screening model. Anomaly scores around zero reflect the average deviation from the normal FT-IR milk fingerprint; scores above zero indicate progressively larger deviations from the normal FT-IR fingerprint.

The remaining spectra were, per wavelength, centered to have zero mean and scaled to unit variance. We then performed a principal component analysis (PCA) on the spectra and extracted the first 16 components that together explained around 95% of the variation in the spectra. Importantly, due to the scaling transformation, the absorption at each wavelength contributed equally to the construction of the eigenvectors. At this time, we also ensured that the four different IR instruments did not emerge as distinct clusters in the latent space. This is important because the mathematical model should not be more sensitive to one instrument than another. Compared to earlier studies, we decided to extract a few more components from the PCA in order to slightly over-fit the data. This was done with the intention to increase the relative contribution of spectral patterns that normally explain only small fractions of the expected variation encountered in milk. Upon encountering atypical spectra, however, variations described by such components could be important. After transforming the spectra to the latent space, we computed the covariance matrix and calculated per spectrum the Mahalanobis distance. The Mahalanobis distance reflects the distance of an individual spectrum to the distribution of all other spectra in the latent space. In the next step, we used PCA to perform an inverse transformation on the spectra in the latent space in order to obtain the reconstructed spectra in the original space. By calculating, across all wavelengths, the root-mean-squared error between the original and reconstructed spectra, we obtained the spectral residuals. In the last step, the Mahalanobis distances and the spectral residuals were each standardized to have zero mean with unit variance before they were summed to a single score per spectrum: the spectrum anomaly score. The higher the spectrum anomaly score, the more a spectrum deviates from all other spectra. In order to reduce the impact of spectral outliers, we performed two iterations using the procedure described above. After each iteration, spectra with the highest 0.1% anomaly scores were removed. The final mathematic model used to identify atypical spectra was based on 344,781 spectra. Figure 1 shows the distribution of anomaly scores of those spectra that were used to construct the mathematical model. 

#### 2.2.2. Classifying Atypical Spectra

Using the final model for untargeted spectra screening, spectrum anomaly scores were computed for the entire main dataset of 5,847,603 spectra. In order to classify spectra as atypical, the anomaly scores have to be thresholded. Because the true prevalence of atypical milk that can be detected with FT-IR is usually unknown, a somewhat arbitrary threshold has to be defined. Given the strict regulations and tight on-farm inspection regimes in the Dutch dairy sector, we assumed one in every thousand (0.1%) milk samples to be atypical—on a monthly basis. In earlier research, a prevalence of up to 1% was sometimes used [6]. Defining the threshold on a monthly basis ensured that we had an equal number of atypical spectra per month over a period of almost three years. This way, a dataset with 5671 atypical spectra was constructed. This dataset was further randomly split into a training (*n* = 4253 spectra) and test dataset (*n* = 1418 spectra).

The 4253 atypical spectra in the training set were centered and scaled using robust statistics (i.e., the median and interquartile range). Data transformations with robust statistics were performed to reduce the impact of spectral outliers. We then used a PCA and extracted the first 24 components that together explained 99.5% of the variance in the spectra. We found the clustering to result in more stable and interpretable results after the spectra had been transformed to the latent space. This is most likely because the PCA reduces the amount of redundancy in the spectra and thereby, relatively speaking, increases the signal-to-noise ratio. We then fitted various Gaussian Mixture Models to the spectral data in the latent space. Gaussian Mixture Models (GMM) are probabilistic generative models and a generalization of the K-means cluster algorithm. In a GMM, multiple multivariate Gaussian distributions are fitted and mixed in such a way that they can generate synthetic data that resemble the actual data as much as possible. The most important hyperparameter of a GMM is the number of components that one has to select a priori. In other words, how many different categories of atypical milk spectra the dataset contains.

The true number of distinct categories cannot be known and depends on many factors. Therefore, many possible cluster solutions exist across different datasets and even within a single dataset. The selection of the optimal solution should be guided by the data (i.e., which solution generalizes to new data) and domain-specific knowledge (which clusters are expected and what do they reflect). This leads to a trade-off between generalizability and interpretability similar to the over- and underfitting trade-off encountered in machine learning problems. Solutions with few clusters may generalize well but may suffer from being too general to be useful (i.e., they underfit the data). Solutions with many clusters, on the other hand, can lead to highly segmented and specific clusters that often fail to generalize to new data (i.e., they overfit the data). We evaluated multiple cluster solutions, each time with a different number of clusters. We considered GMMs with four to twenty components. GMMs were fitted such that each Gaussian component had its own fully-parameterized covariance matrix. This made it possible to fit clusters with different spheroidal shapes. The location parameter of the individual components was initialized using the K-means algorithm. Moreover, each GMM was fitted to the data 50 times and the model that best fitted the data (in terms of the log likelihood) was kept as the final model to be evaluated.

The quality of the results was evaluated by investigating (i) the similarity of spectra within each cluster in relation to the difference between the clusters, (ii) the size of the clusters (i.e., too many small clusters could indicate overfitting, too few large clusters underfitting), (iii) the degree to which clusters mainly contain atypical spectra from only a very small number of farms, (iv) the temporal profile, (v) whether a cluster contains spectra from only one or two FT-IR instruments, and (vi) the compositional milk profile in terms of fat, protein, lactose, urea, freezing point, and milk fat acidity. On the basis of this information, we found that a GMM with 11 components yielded a meaningful cluster solution. However, two clusters were still too similar in terms of the spectra and the compositional milk profile. Moreover, one cluster only contained spectra from two of the four different FT-IR instruments, while the other cluster exclusively contained spectra from the other two FT-IR instruments. In other words, the difference between the two clusters could be mainly attributed to differences between the FT-IR instruments. We therefore decided to group these two clusters together. This resulted in 10 final clusters. 

Although some cluster algorithms can also be used to categorize new data, they are inflexible when it comes to combining multiple clusters into one, such as we did. We therefore followed a more general route and used a dedicated classifier to assign new atypical spectra into one of the identified clusters. We fitted a support vector classifier on the spectra in the latent space using the 10 clusters as class labels. Optimal parameters of the classifier were determined by combining a grid search with 5-fold stratified cross-validation. The cross-validated weighted F1-score of 0.95 indicated excellent classification performance. To obtain an indication about the generalizability of the identified cluster solution, we used the 1418 atypical spectra from the test set. Before predicting their cluster membership, we centered and scaled the spectra and transformed them to the latent space. All data transformations were performed with the parameters calculated from the training set (i.e., median, interquartile range, and eigenvectors). Afterwards, the classifier was used to assign the spectra to their most likely cluster. Generalizability was qualitatively assessed by comparing the distribution of the compositional milk profile between the training and the test dataset.

## 3. Results and Discussion

The goal of the study was an evaluation of the potential of combining untargeted methods for atypical milk spectra screening with cluster algorithms to reveal in what way a milk sample is atypical and what the underlying causes could be. How useful a combined approach is depends on whether atypical milk spectra can be clustered into robust and meaningful categories that can, in turn, be linked to possible root causes. However, identifying meaningful and generalizable clusters in complex data is inherently difficult. Because the true number of distinct clusters and their actual meaning cannot be known a priori, the challenge lies in selecting one out of many possible cluster solutions. In the current study, we tried to balance generalizability and interpretability and found that categorizing atypical milk spectra into ten distinct clusters provided a robust and meaningful description of our dataset.

**Figure 2:**
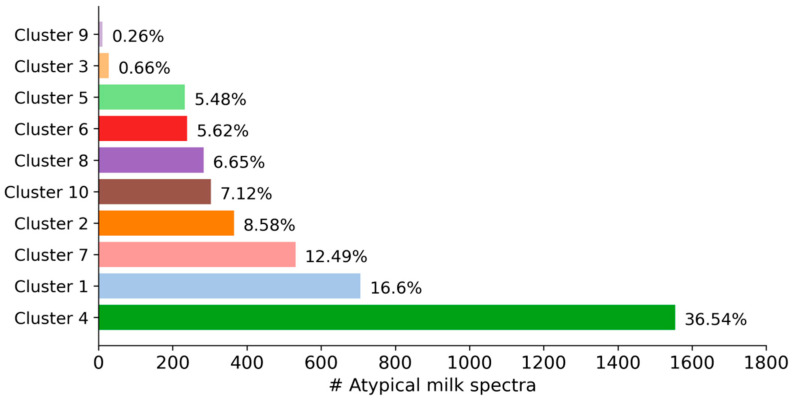
Distribution of the 4253 atypical milk spectra in the 10 clusters.

As can be seen in Figure 2, the size of the clusters varied from 11 milk spectra (0.26% of all spectra) in cluster 9 to 1554 milk spectra (36.54%) in cluster 4. Because some factors that cause milk spectra to be atypical are more likely than others, variability in the size of the clusters is expected. Interestingly, out of 4253 spectra, only 11 milk samples with similar spectral characteristics were necessary to form a distinct cluster. This indicates how sensitive cluster algorithms can be in detecting very small groups of atypical spectra—even within large datasets.

**Figure 3:**
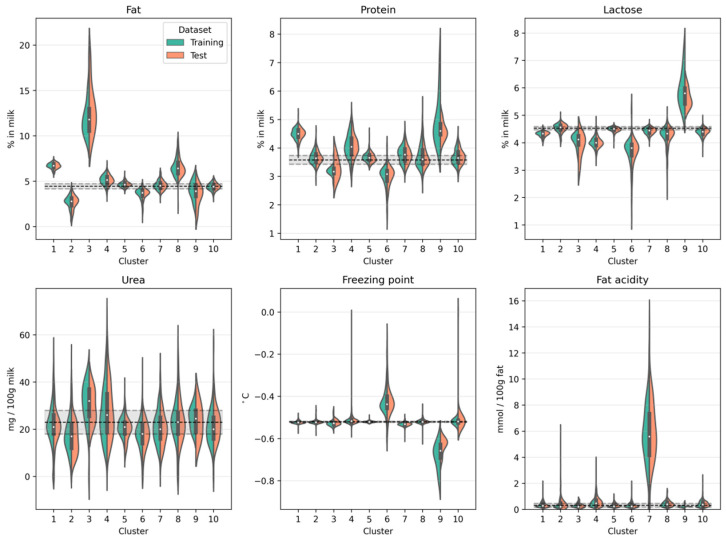
Compositional milk profile per cluster in the training and test datasets. The violin plots show the kernel density estimates for fat, protein, lactose, urea, freezing point, and milk fat acidity for each cluster in the training set (green) and test set (orange). The boxplot contained in each violin describes the minimum, 25, 50, 75%, and maximum value of the training dataset. The horizontal dashed lines and the shaded region correspond to reference values (mean ± 95% confidence interval) calculated from the milk spectra used to develop the untargeted spectra screening model.

Figure 3 shows for both the training dataset and the test dataset the compositional milk profile on a cluster-by-cluster basis. Notice the similarity of the distributions when comparing the training and test dataset. This shows that our cluster solution generalizes to new data and does not merely describe peculiarities in our specific training set. The compositional milk profile also allows for an initial characterization of the clusters so that hypotheses can be made about the underlying root cause. For example, cluster 3 is characterized by an increased fat content of up to 22%. This can be the consequence of insufficient mixing of milk before sampling. Therefore, if untargeted screening methods identify an atypical milk spectrum which is subsequently classified as belonging to cluster 3, it is most likely because the milk has a drastically increased fat content as it was not homogeneously sampled. Cluster 6, on the other hand, is marked by a decreased protein and lactose concentration but an increased freezing point. This is typical for extraneous water in the milk [18]. This can be the result of an influx of water due to improper functioning of valves or an insufficient draining of water residues after cleansing the milking system or the tank. Cluster 7 is marked by a drastic increase in free fatty acids in the milk. This can be the consequence of the lipase activity [19] in sensitive milk (e.g., due to an increased somatic cell count in late lactation cows [20]) or milk that is subject to mechanical strain (e.g., air inclusion in milking systems) [21]. Because increased free fatty acid concentration in the milk can result in rancid flavors of milk products [21,22] and loss of fat in whey during cheese production [23], identifying such atypical milk samples therefore is of direct economic relevance. Finally, cluster 9 is characterized by an increased protein and lactose concentration combined with a decreased freezing point. This could be indicative of adulteration with protein-rich (e.g., milk powder, whey protein isolate) and carbohydrate-based adulterants (e.g., glucose, starch) to increase the apparent concentration of protein and lactose [24]. Individual milk spectra categorized as belonging to cluster 9 could therefore indicate economically motivated adulteration. The ability to identify such cases makes it possible to initiate further target-oriented chemical analyses and contact with the farm for clarification. Although informative, milk spectra can also be atypical for reasons other than those found in the compositional milk profile. For clusters 5 and 10, for instance, the compositional milk profile does not seem to provide relevant diagnostic information. It is therefore also important to focus on the characteristic spectra of the clusters.

**Figure 4:**
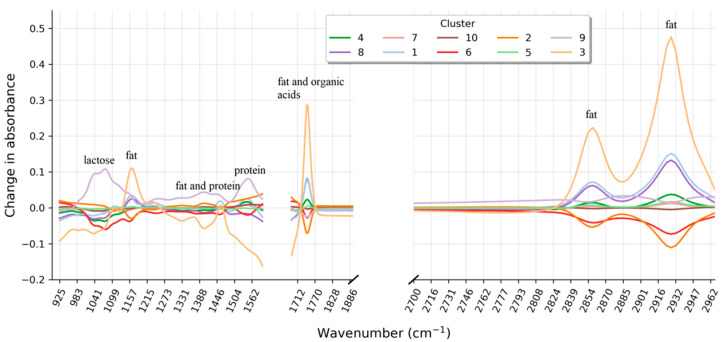
Average change in absorbance per wavenumber for each cluster. The change in absorbance is based on the milk spectra used to develop the untargeted spectra screening model.

Figure 4 gives an illustration of how the average milk spectrum per cluster deviates from a typical milk spectrum. Inspecting the average spectra reveals that the change in absorbance of some clusters mainly occurs in spectral regions that are directly related to the concentration of fat (around 1750 and 2860 cm^−1^), protein (around 1540 cm^−1^), and lactose (around 1040 cm^−1^). Cluster 3, for example, is marked by increased absorption in the regions around 1750 and 2860 cm^−1^ which corresponds to the C = O bonds (carboxyl group) and C-H bonds (ethylene group) characteristic for fat. For cluster 6, on the other hand, decreased absorption around 1540 (H-N-C = O amides) and 1040 cm^−1^ (C-O carbohydrates) correspond to a decreased protein and lactose concentration, respectively. However, some clusters also show a structurally abnormal absorbance profile. A closer look at the spectral region between 2740 and 2970 cm^−1^ reveals an interesting pattern for cluster 9. The spectral absorbance pattern in this region differs from the remaining clusters and the natural variation encountered in typical FT-IR spectra. Similarly, for cluster 2 in the region between 926 and 1100 cm^−1^. Such structural abnormalities can go unnoticed when only the compositional milk profile is analyzed but may provide important information about the nature of the atypical appearance. This information can then be used to generate hypotheses about a possible explanation that can be tested by target-oriented chemical analyses or further exploration on the spot.

**Figure 5:**
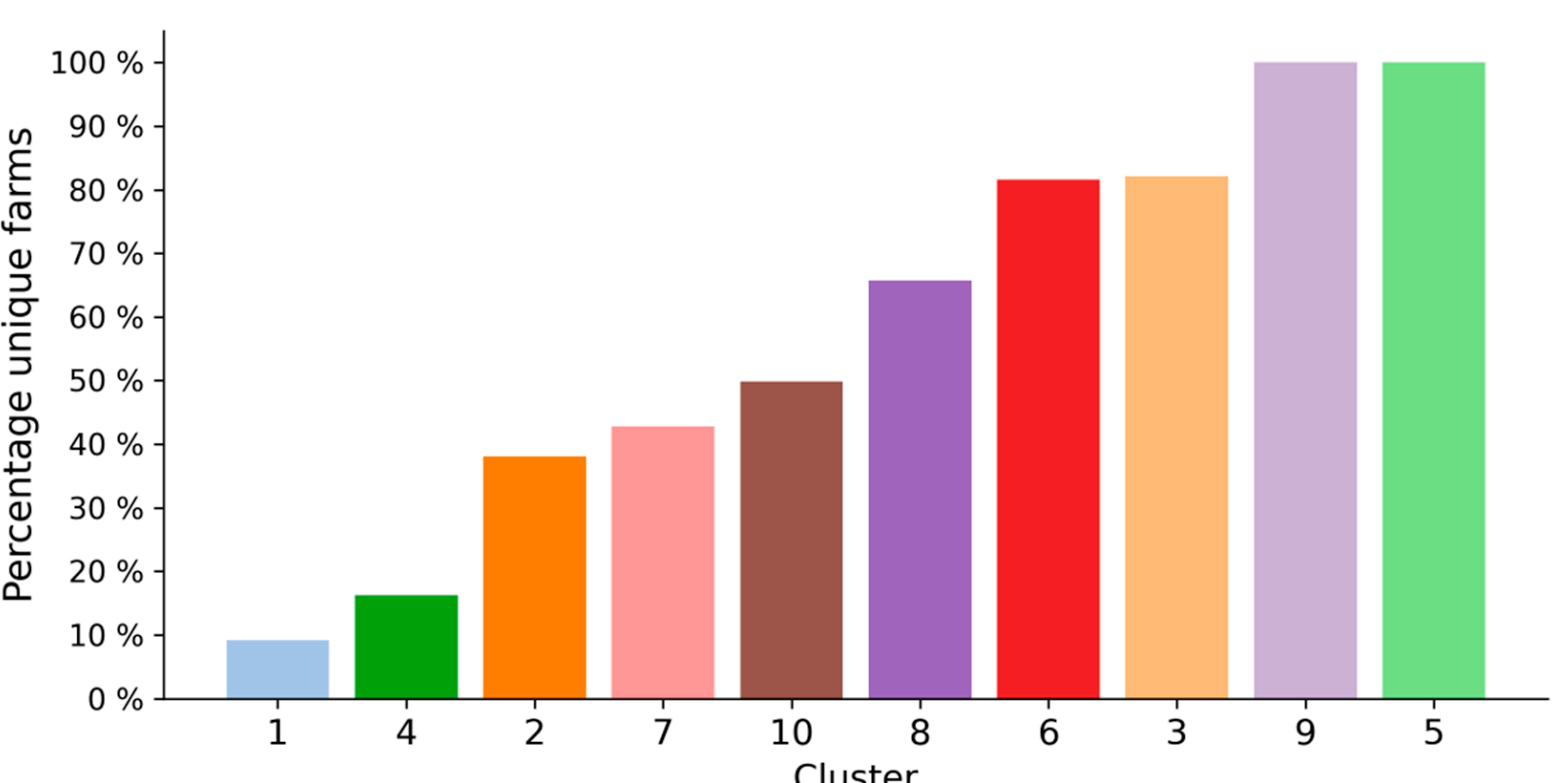
Proportion of unique farms per cluster. The percentages reflect the ratio between how many spectra a cluster contains (i.e., the cluster size) and from how many different farms these spectra are. The lower the proportion of unique farms, the more it indicates that the spectra belong to milk from only few farms.

Describing clusters on the basis of their compositional milk profile and the underlying milk spectra provides important information and allows for an assessment of whether classifying atypical milk spectra is feasible in the first place. However, we believe that an evaluation of the applicability of a cluster solution also depends on important meta-information regarding the atypical spectra. One such source of information relates to the proportion of unique farms in relation to the size of each cluster. This gives an indication of the extent to which the root cause underlying atypical milk spectra within a cluster can be found at many different farms, or only a few farms. As can be seen in Figure 5, most of the distinct clusters have a proportion of unique farms in between 40 and 80%. For example, 43% of all the spectra in cluster 7 belong to different farms. Considering the actual size of the cluster, it can be calculated that the 529 spectra in cluster 7 belong to 227 different farms. This also means that, considering a period of almost 3 years, a farm in cluster 7 will have on average 2.3 atypical spectra in this cluster. This could be indicative of certain factors more prominent at these farms. Knowledge about these factors (e.g., factors leading to increased mechanical strain of milk) could then be used to take preventive measures. We also found two clusters, cluster 1 and cluster 4, where the proportion of unique farms was particularly low (9.33 and 16.34%, respectively). This indicates that the majority of atypical spectra correspond to milk supplies from relatively few farms. In cluster 5 and cluster 9, on the contrary, all spectra belong to different farms. This could either mean that the underlying cause affects farms only incidentally or that the reason is not located at the farms but instead at the laboratory where the FT-IR measurements take place.

**Figure 6:**
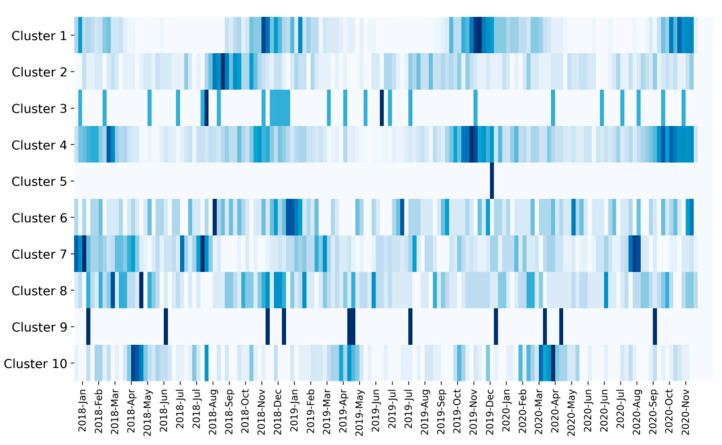
Temporal profile of relative cluster sizes. Shown is for each cluster how many milk samples were identified on a weekly basis over a three-year period, normalized by the relative frequencies. Dark regions reflect periods with a relatively large number of identified milk samples. Bright regions reflect periods with relatively few identified milk samples.

We also analyzed the temporal profile of the clusters. In Figure 6, we visualized per cluster the distribution of milk samples over time. This makes it possible to identify the extent to which a cluster primarily describes milk that is atypical because the underlying root cause has a seasonal profile (such as feeding and variation in temperature). Moreover, it can be useful to know whether the underlying root cause is confined to a temporally isolated event or whether it is more permanent. In our dataset, most of the clusters do not appear to have a seasonal profile but are evenly distributed across the three-year period. This suggests that the clusters are stable in the sense that the underlying root causes exist throughout the year and are likely to be permanent. For example, the few spectra belonging to cluster 9 were discovered evenly throughout a period of three years and each spectrum corresponds to milk from a different farm. This is not indicative of a systematic intentional adulteration, where multiple milk samples from the same farms are expected to be affected over a certain period of time. Nevertheless, evidence suggests that atypical spectra belonging to cluster 9 will also be found in the future, and this may justify further target-oriented analyses concerning these milk samples or the farms they are coming from. We also identified clusters with a seasonal profile. As can be seen in Figure 6, clusters 1, 4, and 10 have a yearly seasonal profile. In cluster 10 the majority of spectra originate from milk samples collected between April and May. This period of the year typically marks the start of the grazing season, which goes along with a transition from silage-based feeding to autonomous grazing. It is well known that this leads to characteristic changes in the milk’s fatty acid profile [25,26]. Because over 80% of the farms in the Netherlands have their cows out in the meadow during the grazing season, natural variation due to grazing is already incorporated in our mathematical model. Cluster 10 could therefore contain milk from farms where the transition to the grazing season leads to particularly drastic changes in the fatty acid profile of the milk—perhaps due to abrupt changes in the food intake. Note that a change in the fatty acid profile does not have to be reflected in the milk’s overall fat percentage. Another two clusters that follow a yearly seasonal profile are cluster 1 and, albeit being less pronounced, cluster 4. Both clusters mainly contain milk samples collected around November and December. This period is likely associated with the transition from autonomous grazing to silage-based feeding at the end of the grazing season. 

Another discovery that can be made from the temporal profiles relates to cluster 5. More than 99% of the spectra in cluster 5 were measured on two consecutive days. We found that all but two spectra were measured on a single FT-IR instrument. This highly suggests that the spectra in cluster 5 are atypical due to temporary instabilities related to the particular FT-IR instrument. For example, multiple reflections inside the layers within the measurement cell of the infrared instrument can produce characteristic artifacts in the spectrum known as fringes [27]. This is an interesting and important discovery. On the one hand, it indicates that such instrument instabilities produce characteristic spectral patterns that can be detected by a cluster algorithm. On the other hand, it shows that such instabilities can lead to drastic changes in the spectrum that go unnoticed when only the milk’s compositional profile or even the raw spectra are analyzed. Moreover, mathematical models used for the calculation of chemical compounds that are present only in small concentrations (e.g., individual fatty acids) can produce invalid results if the instabilities affect spectral features used by these models. In addition, such instabilities can, and in our case did, lead to anomaly scores higher than 99.9% for all other scores in this period. In other words, these spectra were considered as atypical for reasons that are unrelated to the chemical composition of the milk. By analyzing the time-course of anomaly scores on an instrument-by-instrument basis, it is possible to identify periods of instrument instabilities that are reflected as increased anomaly scores. This way, atypical milk spectrum screening can also be used to simultaneously screen FT-IR instruments for instabilities that can lead to systematic changes in milk spectra. Moreover, such instrument instabilities can indicate that the instrument requires maintenance. 

### Implications, Limitations, and Future Outlook

Despite the promising results obtained from our cluster-level analyses, we also acknowledge some limitations. First, it may not always be obvious or even possible to describe what it means for the actual milk if it has a specific atypical absorbance pattern. This is particularly the case for categories characterized by non-trivial absorbance patterns rather than isolated peaks. In our case, we found that clusters 5 and 10 were interesting in this regard. Both clusters could hardly be characterized by inspection of the spectra or their compositional milk profile. Nevertheless, information about why they were atypical could be inferred from the corresponding meta-data. It is therefore possible to identify categories for which an explanation can be given as to why milk spectra are atypical without being able to explain in what way they are atypical. 

Another limitation concerns the classification of new atypical milk spectra. We demonstrated that is possible to identify meaningful categories of atypical milk spectra that also generalize to new datasets. Although generalizability of a cluster solution is crucial, it should be clear that a spectrum is always more similar to one cluster than to another. This means that a milk spectrum will always be assigned to a category, no matter how well it actually fits the category. This can lead to erroneous conclusions about how and why the milk is atypical. Ideally, only those milk spectra that are sufficiently similar to the spectra that were used by the cluster algorithm to identify the categories in the first place should be assigned to a category. Probabilistic generative models such as Gaussian Mixture Models can, theoretically, do exactly that. Since they can be used to generate synthetic data, they can also be used to calculate the probability that a particular milk spectrum can be randomly generated from this model. When the probability for a milk spectrum is particularly low, the model may not be appropriate for categorizing the spectrum. This way, it is also important to keep in mind that a cluster solution will necessarily become outdated over time. This is because the chemical composition of normal milk changes over the years and the reasons that cause milk spectra to be atypical do so too. This requires that the cluster algorithm also be updated. Monitoring, over time, the proportion of atypical spectra that cannot be categorized by the model can give important information about the up-to-datedness of the models. However, more research needs to be done in this area.

A final limitation concerns untargeted milk spectra screening methods in general. Untargeted spectra screening relies on a comparison of an individual milk spectrum with a reference FT-IR milk fingerprint. The reference milk fingerprint represents the gold standard of what is assumed to reflect normal milk. However, milk with a spectrum that deviates from this fingerprint does not automatically reflect poor quality. In fact, even the opposite can be the case. With the use of cluster algorithms and a thorough analysis of the resulting categories, it may be possible to distinguish milk that is atypical for undesired reasons (e.g., adulteration with water) from milk that is atypical for desirable reasons (e.g., particularly good farm management practices or feeding regimes). On the basis of our findings, we believe that a combined approach for atypical spectra screening can serve as a valuable complementary quality assurance tool in routine FT-IR milk analysis. After meaningful and generalizable categories of atypical milk spectra have been identified, new atypical milk spectra can be classified into these categories. Knowledge about the different categories, in terms of what they reflect and what the possible root causes are, can then be used to describe why and in what way a new milk spectrum is atypical. Moreover, our approach is computationally efficient, scalable, and can be implemented in large-scale routine screening for atypical milk. Being able to give an indication about how and why a milk spectrum is atypical is necessary for taking appropriate actions (e.g., rejecting the milk) and is an indispensable prerequisite when contacting the farm for clarification. Our discovery that instrument instabilities can also be responsible for atypical spectra is of particular relevance here. It is important to differentiate between spectra that are atypical because the milk is chemically abnormal or because the FT-IR instrument produces measurement artifacts in the spectrum. This way, atypical spectra screening can be used as a tool for monitoring the quality and authenticity of milk, and for monitoring the FT-IR instruments from which the spectra are obtained and the compositional milk profile is computed.

## 4. Conclusions

When applied to the FT-IR spectra of liquid milk samples, we have shown that a combination of untargeted screening methods and cluster algorithms reveals meaningful and generalizable categories of atypical milk spectra. We demonstrated that information in the spectra (e.g., the compositional milk profile) and meta-data associated with their acquisition (e.g., when and at which instrument) can be used to understand in what way the milk is atypical and how it can be used to form hypotheses about the underlying causes, whether on-farm or in the laboratory. Our combined approach resulted in the identification of ten categories of atypical milk spectra. Some of them could be to linked to increased mechanical strain of the milk, adulteration with extraneous water, or insufficient homogenization during sampling at the farm. Another category could reflect economically motivated adulteration of milk with protein-rich and carbohydrate-based adulterants. We also identified a category that contains spectra that are atypical due to measurement artifacts associated with the infrared instrument. However, more research is needed to confirm these hypotheses. Future studies could also investigate the potential of the described methodology to identify and categorize such measurement artifacts to assess the maintenance status of infrared instruments.

A combined approach in which atypical spectra are detected by untargeted methods and then assigned to categories revealed by cluster algorithms provides important information about how and why a milk spectrum is atypical. This is important for selecting effective and appropriate follow-up actions and greatly extends the practical utility and scope of FT-IR milk screening when used as a complementary tool for quality assurance in the dairy.

## Data Availability

Not applicable.
